# Scion genotype dominates drought tolerance in grafted *Coffea spp.* seedlings under severe water stress

**DOI:** 10.3389/fpls.2026.1835057

**Published:** 2026-05-20

**Authors:** Zhiwei Ding, Abdul Majeed Baloch, Li Gong, Abdul Wahid Baloch, Jalil Ahmed, Fayyaz Maqbool, Sheharyar Ali, Yunping Dong, Xuejun Li, Weifeng Li, Zhenjiang Lv

**Affiliations:** 1 College of Tropical Crops, Yunnan Agricultural University, Puer, China; 2 Department of Plant Breeding and Genetics, Sindh Agriculture University, Tando Jam, Pakistan; 3 Department of Entomology, Sindh Agriculture University, Tando Jam, Pakistan; 4 Department of Horticulture, Sindh Agriculture University, Tando Jam, Pakistan; 5 Yunnan Key Laboratory of Coffee, Yunnan Agricultural University, Puer, China

**Keywords:** *Coffea*, drought tolerance, interspecific grafting, intraspecific grafting, scion genotype

## Abstract

**Introduction:**

Drought is a major abiotic stress limiting coffee production globally. While grafting improves abiotic stress tolerance, the relative contributions of scion and root stock to drought resistance in intraspecific and interspecific grafted *Coffea* seedlings remain unclear across different stress intensities.

**Methods:**

We hypothesized that scion genotype dominates drought responses under severe water stress, whereas rootstock effects prevail under normal irrigation. To test this, we conducted a 120-day pot experiment using daily weighing and replenishment, with three treatments: normal supply (18-25% soil water), mild drought (15-17%), and severe drought (10-12%). Two *C. canephora* scions (‘Dafeng No.1’,‘Reyan No.5’) were grafted onto intraspecific (‘Reyan No.2’) and interspecific (‘Charlie No.16’) rootstocks.

**Results:**

‘Reyan No.5’ scion conferred superior drought tolerance under severe stress via stronger membrane stability (lower malondialdehyde, MDA), higher osmotic adjustment, and more stable antioxidant defense (super oxide dismutase, SOD; peroxidase, POD; catalase, CAT) .‘Dafeng No.1’ showed higher water use efficiency (WUE) under mild stress but suffered greater oxidative damage. Principal component analysis confirmed scion genotype explained 44. 78% of total physiological variation.

**Discussion:**

These findings clarify scion dominance in severe drought tolerance and guide drought-resistant coffee grafting selection.

## 1 Introduction

With global warming and frequent natural disasters, drought stress has become one of the major abiotic stresses for plants (Rivero et al., 2003; Luo, 2010; Chaeikar et al., 2020). There are a series of morphological, physiological, biochemical, and molecular changes in plants under water deficit conditions (Ahmed et al., 2009; Liu et al., 2014; Kumar et al., 2017). Morphologically, *Coffea* species exhibit conserved adaptive strategies under water deficit, including reduced vegetative growth, smaller leaf area, thicker cuticle, and enhanced root elongation. These changes minimize transpirational water loss and improve soil water exploration capacity (Dias et al., 2007). Physiologically, water deficit reduces CO_2_ uptake in coffee plants, inhibiting leaf photosynthesis. This further disrupts the electron transport chain and induces oxidative stress (Pinheiro and Chaves, 2011). Furthermore, molecular studies across diverse plant species have uncovered conserved and functionally relevant mechanisms underlying drought stress tolerance. Zhang et al. (2011) demonstrated that heterologous expression of the *Arabidopsis thaliana* ABP9 gene mitigates oxidative damage and confers enhanced drought tolerance in transgenic plants. In tobacco (*Nicotiana tabacum*), overexpression of the GbMPK3 gene significantly elevates the activities of antioxidant enzymes and improves drought resistance by augmenting reactive oxygen species (ROS) scavenging capacity (Long et al., 2014). The genus *Coffea* comprises perennial evergreen shrubs and small trees in the Rubiaceae family. Among these species, *Coffea arabica*, *Coffea canephora*, and *Coffea liberica* are globally significant tropical cash crops, whose production sustains the livelihoods of millions of people across tropical and subtropical regions (Kufa, 2012). Drought stress, which disrupts the normal growth and developmental processes of coffee plants, has long been recognized as a primary abiotic limiting factor for coffee production (Praxedes et al., 2006). In recent decades, increasing water scarcity and more frequent severe drought events driven by global climate change have further exacerbated this threat to global coffee supplies (León-Burgos et al., 2022). This persistent drought stress imposes multifaceted challenges on coffee cultivation. It degrades suitable growing habitats, causes significant declines in coffee bean yield and quality, increases the incidence of pests and diseases, and ultimately leads to substantial economic losses for producers (Silva et al., 2022). Against this background, developing drought-tolerant coffee varieties that can maintain vigorous growth and stable yields under water-limited conditions has become an urgent priority for coffee breeding programs worldwide (Yang et al., 2022).

Grafting is a well-established traditional asexual propagation technique that enhances plant growth and development, increases crop yield, improves fruit flavor and nutritional quality, and confers broad-spectrum abiotic stress tolerance. These benefits arise from its unique ability to retain and combine the complementary superior traits of both scion and rootstock (Sánchez-Rodríguez et al., 2014; Thomas and Frank, 2019; Ellenberger et al., 2021). To date, grafting has been extensively applied in agricultural and horticultural production systems worldwide (Vanderklein et al., 2007; Greenwood et al., 2010; Tombesi et al., 2010; Goldschmidt, 2014). Accumulating evidence demonstrates that compatible graft combinations significantly enhance drought tolerance in diverse plant species, including polyploid mulberry (Hui et al., 2024), grape (Jiao et al., 2023), cucumber (Shehata et al., 2022), poplar (Han et al., 2019), and tomato (Fullana-Pericàs et al., 2018). While substantial progress has been made in understanding grafting-mediated drought tolerance in model and major crop species, the underlying mechanisms in coffee remain poorly characterized. Recent studies have focused increasing attention on the physiological and biochemical responses of diverse *Coffea* genotypes to drought stress, leading to several key insights. First, drought stress triggers rapid stomatal closure and a marked reduction in net photosynthetic rate in coffee leaves, and this photosynthetic inhibition is attributed to combined stomatal and photochemical limitations (de Souza et al., 2020). Second, prolonged drought exposure not only induces significant alterations in soluble sugar profiles but also causes irreversible declines in leaf water potential (Tounekti et al., 2018). Furthermore, accumulation of galactitol and raffinose family oligosaccharides (RFOs) in nutritive tissues including leaves and endosperm has been shown to play a critical role in coffee drought tolerance (Ivamoto et al., 2017). While previous studies have confirmed the drought tolerance of grafted *Coffea* seedlings, the relative contributions of scion and rootstock to drought resistance remain unclear, especially the differential responses of intraspecific and interspecific grafting combinations under different water deficit intensities.

To fill this critical research gap, the present study employed intraspecific and interspecific grafted coffee seedlings as experimental materials. We systematically assessed the drought resistance profiles of different graft combinations under a series of water stress conditions through comprehensive measurements of photosynthetic gas exchange parameters, malondialdehyde content, osmotic adjustment substances, and antioxidant enzyme activities. This study aimed to: (1) investigate the dynamic changes in photosynthetic parameters, membrane peroxidation, osmotic regulators, and antioxidant enzyme activities of intraspecific and interspecific grafted *Coffea* seedlings under different arid conditions levels; (2) compare the physiological differences between intraspecific and interspecific grafted seedlings in response to drought; (3) clarify the relative contributions of scion and rootstock genotypes to the drought resistance of grafted seedlings. We further hypothesized that scion genotype would play a dominant role in regulating drought tolerance under severe water stress, while rootstocks would have a greater impact under normal water supply. Our findings provide theoretical support for the vegetative propagation and rational selection of drought-resistant coffee graft combinations.

## 2 Materials and methods

### 2.1 Plant materials and pretreatments

The plant materials for this study were ‘Dafeng No.1’ (*Coffea canephora* Pierre ex A. Froehner cv. Dafeng No.1, scion), ‘Reyan No.5’ (*Coffea canephora* Pierre ex A. Froehner cv. Reyan No.5, scion), ‘Charlie No.16’ (*Coffea liberica* Bull ex Hiern cv. No.16, rootstock) and ‘Reyan No.2’ (*Coffea canephora* Pierre ex A. Froehner cv. Reyan No.2, rootstock). All those materials were from the Institute of Spices and Beverages of the Chinese Academy of Tropical Agricultural Sciences. The four coffee genotypes used in this study were selected based on their agronomic importance and widespread cultivation in Hainan Province, China, which is one of the main coffee-producing regions in the country. ‘Dafeng No.1’ and ‘Reyan No.5’ are the two most widely planted *Coffea canephora* scion cultivars in Hainan, with high yield and good bean quality. Previous preliminary observations suggested that ‘Reyan No.5’ may have better drought tolerance than ‘Dafeng No.1’, but no systematic physiological study has been conducted to confirm this. ‘Reyan No.2’ is the most commonly used intraspecific rootstock for *C. canephora* propagation, with good graft compatibility and growth performance. ‘Charlie No.16’ is a *C. liberica* cultivar with a deep and well-developed root system, which is expected to improve water and nutrient uptake under stress conditions. It has been increasingly used as an interspecific rootstock for coffee in recent years.

In February 2018, fresh fruits were collected from healthy mother plants of ‘Reyan No. 2’ and ‘Charlie No. 16’ for seed production, and the seeds were sown in sand beds in a solar greenhouse. The experiment was conducted in the same solar greenhouse under strictly controlled environmental conditions: a constant temperature of 25 ± 3°C, relative humidity of 70 ± 5%, and midday photosynthetically active radiation of approximately 43,000 lux throughout the entire experimental period. All pots were randomly placed on the same elevated bench, and their positions were re-randomized weekly to eliminate any potential micro-environmental gradients. In August 2018, seedlings with uniform vigor were transplanted into nursery pots and cultured until March of the following year. The nursery pots were 26 cm in diameter and 30 cm in height. The growth substrate consisted of topsoil and cow manure at a ratio of 85:15 (w/w), with pH 6.5 ± 0.2, organic matter content 28.6 g kg^-1^, total nitrogen 1.52 g kg^-1^, available phosphorus 68.3 mg kg^-1^, and total substrate weight of 16.31 kg.

In March 2019, scions were collected from healthy mother plants of ‘Dafeng No. 1’ and ‘Reyan No. 5’ and grafted onto ‘Reyan No. 2’ and ‘Charlie No. 16’ using the single-bud side-grafting (ventral budding) method. Briefly, rootstocks with a stem diameter of ≥0.8 cm were selected, and a rectangular incision (4 cm in length) was made 10 cm above the soil line, penetrating to the cambium layer. Single-bud scions (4 cm long, with one full axillary bud) were cut from semi-lignified shoots, their lower ends trimmed to a 45° bevel and inserted into the rootstock incisions to ensure at least one side of the cambium was aligned. Graft unions were tightly wrapped with 1 cm wide white plastic tape, leaving the bud exposed. Binding tapes were removed 50 days post-grafting, and rootstocks were cut back 3 cm above the graft union 10 days later. Fifty grafted seedlings were prepared for each intraspecific and interspecific combination. Intraspecific grafted combinations (‘Dafeng No. 1’/’Reyan No. 2’, ‘Reyan No. 5’/’Reyan No. 2’) were labeled C1 and C5, respectively, based on the rootstock species *C. canephora*. Interspecific grafted combinations (‘Dafeng No. 1’/’Charlie No. 16’, ‘Reyan No. 5’/’Charlie No. 16’) were labeled L1 and L5, respectively, based on the rootstock species *C. liberica*. After grafting, the overall graft take rate exceeded 85%. At 9 months post-grafting, only uniformly grown, fully healed grafted seedlings with no signs of graft incompatibility or abnormal growth were selected for the drought stress experiment. All selected seedlings had well-developed root systems and intact graft unions with no visible swelling or necrosis.

Before the start of the drought stress treatment, all seedlings were fully watered to field capacity for 7 consecutive days to ensure uniform soil water content. The target soil water content ranges were established based on the field capacity of the substrate (25% by weight). Soil water content was measured daily at 18:00 by weighing the entire pot (substrate + seedling + pot). Deionized water was added immediately to replenish the water lost through transpiration and evaporation, maintaining the soil water content within the target range. The drought stress treatment was continued for 120 consecutive days, and all measurements were performed at the end of the treatment period.

A completely randomized design (CRD) with a 4 × 3 factorial arrangement was adopted in this experiment. The two factors were: (1) grafting combination (4 levels: C1, C5, L1, L5) and (2) soil water content (3 levels: normal supply, mild drought, severe drought), resulting in a total of 12 treatment combinations. Interspecific grafting normal watering (L1-I, L5-I, 18% ≤ soil water content ≤ 25%), mild water stress (L1-II, L5-II, 15% ≤ soil water content ≤ 17%), and severe water stress (L1-III, L5-III, 10% ≤ soil water content ≤ 12%); and intraspecific grafting normal watering (C1-I, C5-I, 18% ≤ soil water content ≤ 25%), mild water stress (C1-II, C5-II, 15% ≤ soil water content ≤ 17%), and severe water stress (C1-III, C5-III, 10% ≤ soil water content ≤ 12%). A total of 108 grafted seedlings were used in this experiment. Each of the 12 treatment combinations included 3 independent biological replicates, with 3 uniformly grown seedlings per biological replicate. For all physiological and photosynthetic measurements, the average value of the 3 seedlings in one biological replicate was used as the replicate data. No technical replicates were performed in this study. All statistical analyses were based on n=3 biological replicates per treatment.

### 2.2 Determination of photosynthetic parameters

After 4 months of continuous water stress treatment, photosynthetic parameters were measured on fully expanded functional leaves (the 3rd pair of leaves counted from the shoot tip) using a Li-6400 portable photosynthesis system (LI-COR Inc., Lincoln, NE, USA) between 9:00 and 10:00 AM on sunny days. During measurements, the instrument was set to the following standard conditions consistent with the greenhouse environment: reference CO_2_ concentration of 400 μmol mol^-1^ (ambient atmospheric CO_2_ level), photosynthetically active radiation (PAR) of 1200 μmol m^-2^ s^-1^ (light saturation point for *C. canephora* leaves), leaf chamber temperature of 25 °C, and airflow rate of 500 μmol s^-1^. One leaf was measured per seedling, and three uniformly grown seedlings were measured for each biological replicate. The average value of the three leaves was used as the final data point for that biological replicate. The measured parameters included net photosynthetic rate (*P*n), stomatal conductance (*G*s), intercellular CO_2_ concentration (*C*i), and transpiration rate (*T*r). Water use efficiency (WUE) was calculated as the ratio of *P*n to *T*r (DaMatta et al., 2002).

### 2.3 Relative conductivity determination

The 4th pair of fully expanded leaves (counting from the tip) of each treatment were collected and cut into 0.2 cm^2^ segments. The leaves were rinsed three times in deionized water and then placed in tubes with 20 mL of deionized water, followed by shaking for about 24 h. The conductivity was measured using a conductivity meter (sr60ic, USA) and recorded as C1. The samples were autoclaved at 120°C for 30 min to completely rupture the cell membranes. After the tubes cooled to room temperature, the electrical conductivity of the solution containing the killed tissues was measured and recorded as C2. Relative conductivity (RC) is calculated as C1/C2.

### 2.4 Measurement of physiological indexes

The 3rd and 4th fully expanded leaves from the shoot tip were sampled from each seedling, immediately frozen in liquid nitrogen, and stored at -80 °C until analysis. Malondialdehyde (MDA), proline (Pro), soluble sugar (SS), superoxide dismutase (SOD), peroxidase (POD), catalase (CAT), and glutathione (GSH) were determined using commercial assay kits (Nanjing Jiancheng Bioengineering Institute, Nanjing, China). All procedures were performed strictly according to the manufacturer’s instructions with no modifications. Briefly, MDA was measured by the thiobarbituric acid method to reflect lipid peroxidation (Heath and Packer, 1968). Proline was determined using ninhydrin colorimetry (Bates et al., 1973). Soluble sugar content was quantified via the anthrone colorimetric method (Yemm and Willis, 1954). SOD, POD, and CAT activities were measured using respective substrate colorimetric methods (Chance and Maehly, 1955; Giannopolitis and Ries, 1977). GSH content was determined using the dithiobis nitrobenzoic acid (DTNB) method (Griffith, 1980). These methods are widely used in coffee drought physiology studies and follow standard plant physiological biochemical protocols.

### 2.5 Data statistics and analysis

Two-way analysis of variance (ANOVA) was used to test the effects of grafting combination, water stress, and their interaction on each physiological index. Multiple comparisons were performed using Tukey’s honestly significant difference (HSD) test at *p* < 0.05. The Pearson correlation coefficient method was utilized to analyze the correlation between indexes. Principal component analysis (PCA) were conducted to illustrate the contribution of each index. Before PCA analysis, all data were standardized using the Z-score method to eliminate the influence of different units. PCA was performed using the R packages vegan (v2.6-4) and factoextra (v1.0.7), with R 4.3.2 software.

## 3 Results

### 3.1 Effects of grafting combination and water treatment on photosynthetic parameters

**Figure 1:**
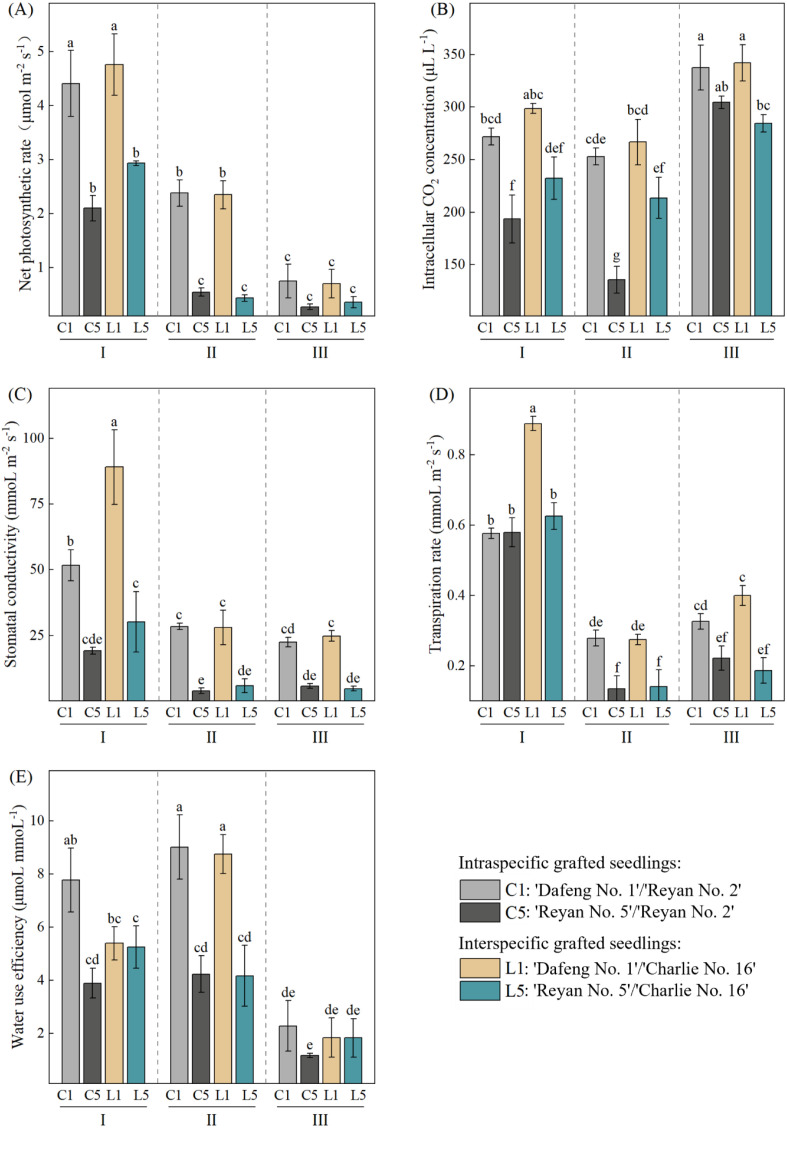
Changes in photosynthetic parameters of four grafting combinations under different water treatments. **(A)** net photosynthetic rate (*P*n); **(B)** intercellular CO_2_ concentration (*C*i); **(C)** stomatal conductance (*G*s); **(D)** transpiration rate (*T*r); **(E)** water use efficiency (WUE). I, normal irrigation; II, mild water stress; III, severe water stress. Data are presented as mean ± standard deviation (n = 3). Two-way ANOVA followed by Tukey HSD test was used to determine significant differences (*p* < 0.05). Different lowercase letters indicate significant differences among treatments (*p* < 0.05).

Two-way ANOVA showed that grafting combination (G), water treatment (W), and their interaction (G × W) all had significant effects on every photosynthesis parameter (**Table 1**). This means both grafting method and water condition change how plants photosynthesize, and these two factors work together rather than acting separately. For net photosynthetic rate (*P*n), water treatment had the strongest effect (F = 267.67, *p* < 0.001). As water stress got worse, *P*n dropped in all grafting combinations. Under normal watering (I), the interspecific graft L1 had the highest *P*n at 4.76 μmol m^-2^ s^-1^. The intraspecific graft C1 came next at 4.41 μmol m^-2^ s^-1^. Both were significantly higher than L5 and C5 (*p* < 0.05). Different rootstocks with the same scion showed no significant differences (*p* > 0.05). Under mild water stress (II), *P*n fell sharply in all combinations. But combinations with ‘Dafeng No.1’ as the scion (C1 and L1) still had higher *P*n than those with ‘Reyan No.5’ as the scion (C5 and L5). Under severe water stress (III), the differences between combinations became much smaller. C1 and L1 showed no significant difference, and C5 and L5 showed no significant difference either (**Figure 1A**). This suggests that under normal and mild drought conditions, the photosynthetic traits of the scion itself are the main factor that determines photosynthetic capacity in grafted coffee seedlings. Intercellular CO_2_ concentration (*C*i) moved in the opposite direction from *P*n. As water stress increased, *C*i went up overall. Under severe water stress (III), L1 had the highest Ci at 342.08 μL L^-1^. C1 was close behind at 337.75 μL L^-1^. Both were significantly higher than C5 and L5 (*p* < 0.05). Under normal watering (I), C5 had the lowest *C*i at 193.58 μL L^-1^, which was significantly lower than all other combinations (*p* < 0.05) (**Figure 1B**). Stomatal conductance (*G*s) and transpiration rate (*T*r) followed the same pattern as *P*n. Both dropped as water stress got worse. Under normal watering, L1 and C1 with ‘Dafeng No.1’ as the scion had higher *G*s than L5 and C5 with ‘Reyan No.5’ as the scion. Under severe water stress, all combinations showed low *G*s and *T*r levels, and some combinations showed no significant differences (*p* > 0.05) (**Figures 1C, D**). According to Farquhar’s photosynthesis limitation theory (Farquhar et al., 1980), when *P*n and *G*s both drop and *C*i also drops, stomatal limitation is the main factor. When *P*n and *G*s drop but *C*i rises, non-stomatal limitation is the main factor. In this study, *P*n, *G*s, and *T*r all dropped together as water stress got worse, but *C*i first dropped then rose. This suggests that stomatal limitation was the main factor under mild water stress. Stomata closed and CO_2_ supply dropped. Under severe water stress, it shifted to non-stomatal limitation. This was mainly caused by damage to the photosynthetic machinery and weaker CO_2_ fixation ability in the Calvin cycle. All graft combinations showed lower water use efficiency (WUE) as water stress got worse. Under normal watering and mild stress, combinations with ‘Dafeng No.1’ as the scion had higher WUE than those with ‘Reyan No.5’ as the scion. Under severe water stress, the differences between combinations were small (**Figure 1E**). The reason is clear: under mild stress, ‘Dafeng No.1’ combinations achieved higher WUE by keeping their *P*n high and lowering their *T*r. Under severe water stress, the photosynthetic systems of all grafted seedlings were badly damaged. Their *P*n dropped sharply, so the differences in WUE between different combinations became much smaller. Overall, combinations with ‘Dafeng No.1’ as the scion (C1 and L1) performed better under normal watering and mild water stress. Combinations with ‘Reyan No.5’ as the scion (C5 and L5) were more sensitive to water stress.

**Table 1:** Results of two-way ANOVA for the effects of grafting combination (G), water treatment (W), and their interaction (G × W) (F-values) on photosynthetic parameters.

Photosynthetic parameters	G	W	G×W
Net photosynthetic rate	49.31^***^	267.67^***^	6.84^***^
Intracellular CO_2_ concentration	63.57^***^	127.49^***^	6.90^***^
Stomatal conductivity	78.46^***^	113.97^***^	12.53^***^
Transpiration rate	86.42^***^	740.92^***^	14.85^***^
Water use efficiency	27.59^***^	106.42^***^	7.45^***^

Significant level: “*” represents *p* < 0.05; “**” represents *p* < 0.01; “***” represents *p* < 0.001.

### 3.2 Effects of grafting combination and water treatment on membrane damage

**Figure 2:**
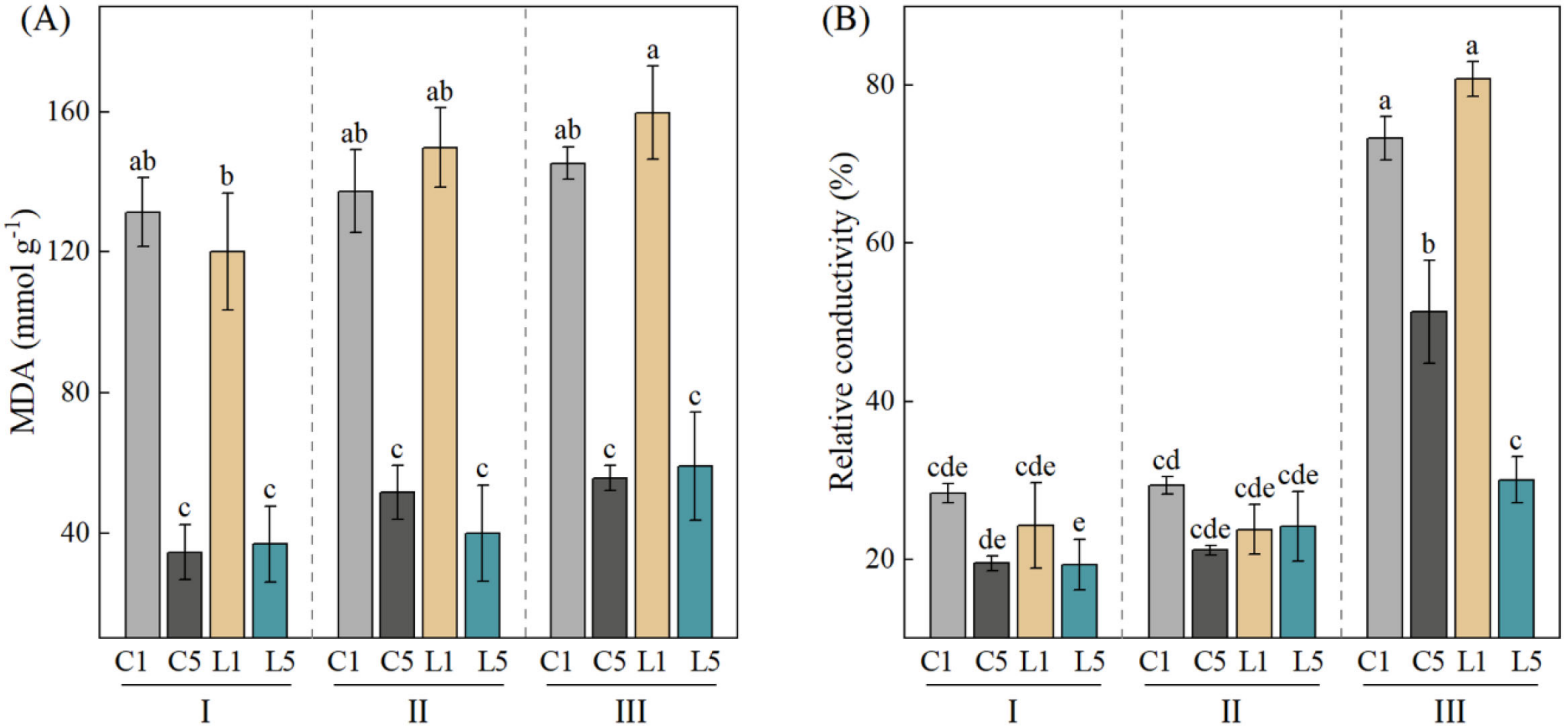
Changes in malondialdehyde (MDA) content and relative conductivity of four grafting combinations under different water treatments. I, normal irrigation; II, mild water stress; III, severe water stress. C1: 'Dafeng No.1'/'Reyan No.2' (intraspecific grafting); C5: 'Reyan No.5'/'Reyan No.2' (intraspecific grafting); L1: 'Dafeng No.1'/'Charlie No.16' (interspecific grafting); L5: 'Reyan No.5'/'Charlie No.16' (interspecific grafting). Data are presented as mean ± standard deviation (n = 3). Two-way ANOVA followed by Tukey HSD test was used to determine significant differences (*p* < 0.05). Different lowercase letters indicate significant differences among treatments (*p* < 0.05).

Two-way ANOVA revealed that grafting combination (G) was the dominant factor affecting MDA content (F = 212.79, *p* < 0.001), while water treatment (W) had a smaller but still significant effect (F = 14.08, *p* < 0.001). Notably, the interaction between grafting combination and water stress (G×W) was not significant for MDA (*p* > 0.05), indicating that the effect of grafting combination on membrane stability was consistent across all water conditions. For relative conductivity, both G, W and their interaction were highly significant (*p* < 0.001) (**Table 2**). MDA is the final product of membrane lipid peroxidation. Higher MDA levels mean more damage to cell membranes. In this study, MDA levels rose in all grafting combinations as drought got worse. This shows that drought gradually damages the cell membrane system. Under all water conditions, combinations with ‘Dafeng No.1’ as the scion (C1 and L1) had significantly higher MDA levels than combinations with ‘Reyan No.5’ as the scion (C5 and L5) (*p* < 0.05). Under the same water treatment, combinations using the same scion showed no significant differences (*p* > 0.05). Combinations using the same rootstock showed significant differences (*p* < 0.05). This suggests that MDA content depends more on rootstock than on scion (**Figure 2A**). Relative conductivity shows how much cell membranes are damaged. Higher relative conductivity means more membrane damage and worse drought resistance. **Figure 2B** shows that relative conductivity rose sharply in grafted coffee as water stress got worse. Under severe water stress (III), the interspecific graft L1 had the highest relative conductivity. C1 came next. Both were significantly higher than C5 and L5 (*p* < 0.05). Under normal watering (I) and mild water stress (II), the differences between combinations were small. In summary, grafting combinations with ‘Reyan No.5’ as the scion (C5 and L5) showed lower membrane lipid peroxidation and lighter cell membrane damage under all water conditions. They had better membrane stability and drought resistance than combinations with ‘Dafeng No.1’ as the scion. Among them, the interspecific graft L5 (‘Reyan No.5’/’Charlie No.16’) had the lowest MDA content and relative conductivity under severe water stress. It showed the strongest resistance to membrane oxidative damage.

**Table 2:** Results of two-way ANOVA on the effects of grafting combination (G), water treatment (W), and their interaction (G × W) (F-values).

Treatment	G	W	G×W
Malondialdehyde	212.79^***^	14.08^***^	1.21
Relative conductivity	70.75^***^	438.53^***^	38.46^***^

Significant level: “*” represents *p* < 0.05; “**” represents *p* < 0.01; “***” represents *p* < 0.001.

### 3.3 Effects of grafting combination and water treatment on osmotic adjustment substances

**Figure 3:**
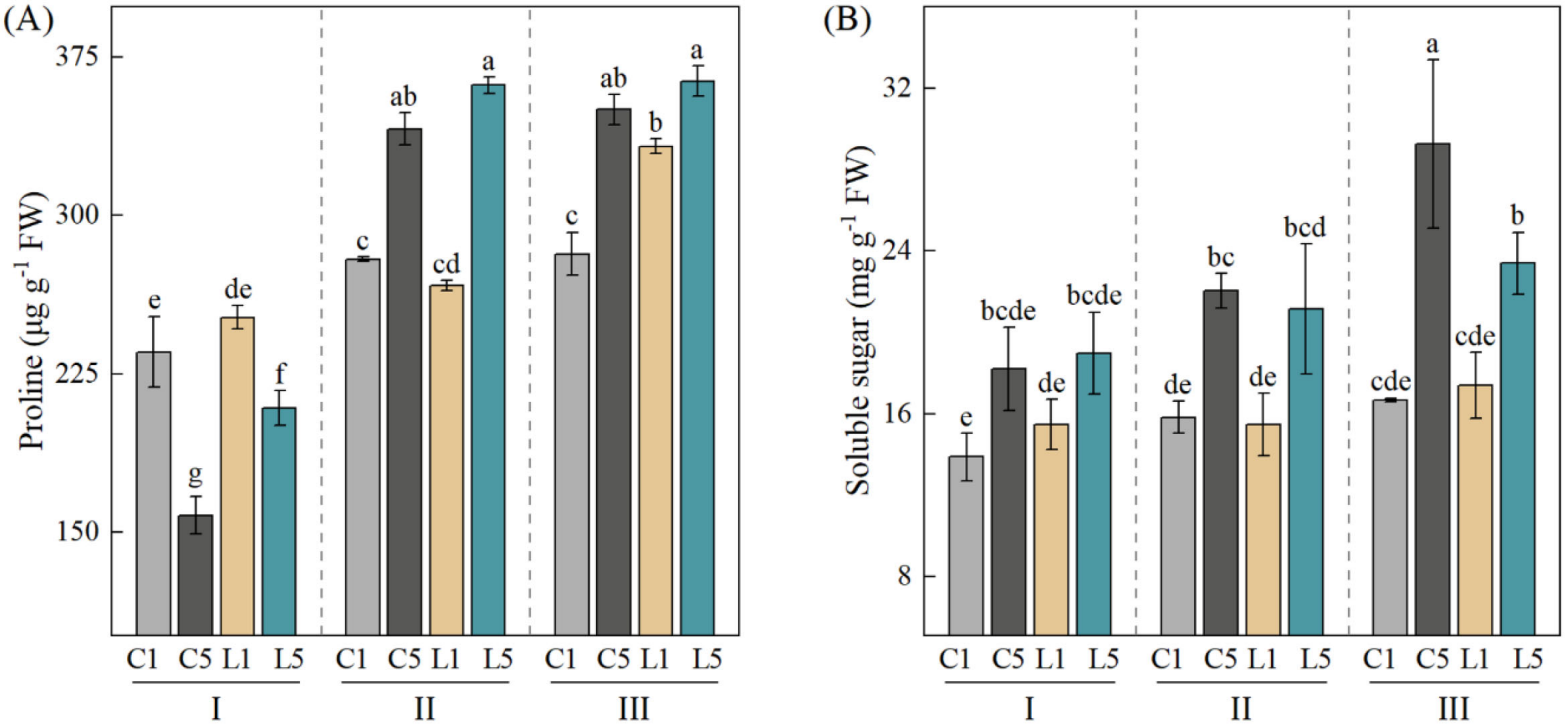
Changes in proline and soluble sugar contents of four grafting combinations under different water treatments. I, normal irrigation; II, mild water stress; III, severe water stress. C1: 'Dafeng No.1'/'Reyan No.2' (intraspecific grafting); C5: 'Reyan No.5'/'Reyan No.2' (intraspecific grafting); L1: 'Dafeng No.1'/'Charlie No.16' (interspecific grafting); L5: 'Reyan No.5'/'Charlie No.16' (interspecific grafting). L5: ‘Reyan no.5’/’Charlie no.16’ (interspecific grafting). Data are presented as mean ± standard deviation (n = 3). Two-way ANOVA followed by Tukey HSD test was used to determine significant differences (*p* < 0.05). Different lowercase letters indicate significant differences among treatments (*p* < 0.05).

Osmotic adjustment represents one of the critical physiological mechanisms enabling plants to cope with drought stress. Two-way ANOVA revealed that grafting combination (G), water treatment (W), and their interaction (G×W) all significantly influenced proline and soluble sugar contents. Water treatment exerted the strongest effect on proline accumulation (F = 764.21, *p* < 0.001), whereas grafting combination showed the greatest impact on soluble sugar content (F = 32.88, *p* < 0.001). The G×W interaction significantly affected soluble sugar levels (*p* < 0.05) (**Table 3**). Proline content increased across all grafting combinations as water stress intensified. Under well-watered conditions, combinations grafted with ‘Dafeng No.1’ (C1, L1) maintained significantly higher proline levels than those with ‘Reyuan No.5’ (C5, L5) (*p* < 0.05). This pattern reversed under mild water stress, with ‘Reyan No.5’-grafted combinations (C5, L5) surpassing ‘Dafeng No.1’ combinations (C1, L1) in proline content (*p* < 0.05). At severe water stress, between-group differences narrowed considerably; notably, the interspecific graft L5 achieved the highest proline concentration (**Figure 3A**). Water stress also elevated soluble sugar content in all combinations. Under well-watered and mild stress conditions, soluble sugar levels varied little among grafting combinations, with no significant differences between mild stress and control treatments for any group (*p* > 0.05). Severe stress, however, ‘Reyan No.5’-grafted combinations (C5, L5) accumulated significantly more soluble sugars than ‘Dafeng No.1’ combinations (C1, L1) (*p* < 0.05), with the intraspecific graft C5 reaching peak values (**Figure 3B**). This finding aligns with the significant G×W interaction observed for soluble sugars (*p* < 0.05) (**Table 3**), pointing to distinct drought-responsive accumulation patterns across grafting combinations. Overall, grafting combinations C5 and L5 showed much stronger ability to build up proline and soluble sugars under water stress, especially under severe drought. This suggests that the scion ‘Reyan No.5’ has better osmotic adjustment capacity. It can maintain cell turgor and stabilize cell structure by accumulating more osmotic adjustment substances under drought stress, which helps improve drought resistance in grafted seedlings.

**Table 3:** Results of two-way ANOVA on the effects of grafting combination (G), water treatment (W), and their interaction (G×W) on proline and soluble sugar contents (F-values).

Treatment	G	W	G×W
Proline	51.09^***^	764.21^***^	95.15^***^
Soluble sugar	32.88^***^	19.76^***^	3.53^*^

Significant level: “*” represents *p* < 0.05; “**” represents *p* < 0.01; “***” represents *p* < 0.001.

### 3.4 Effects of grafting combination and water treatment on antioxidant enzymes

**Figure 4:**
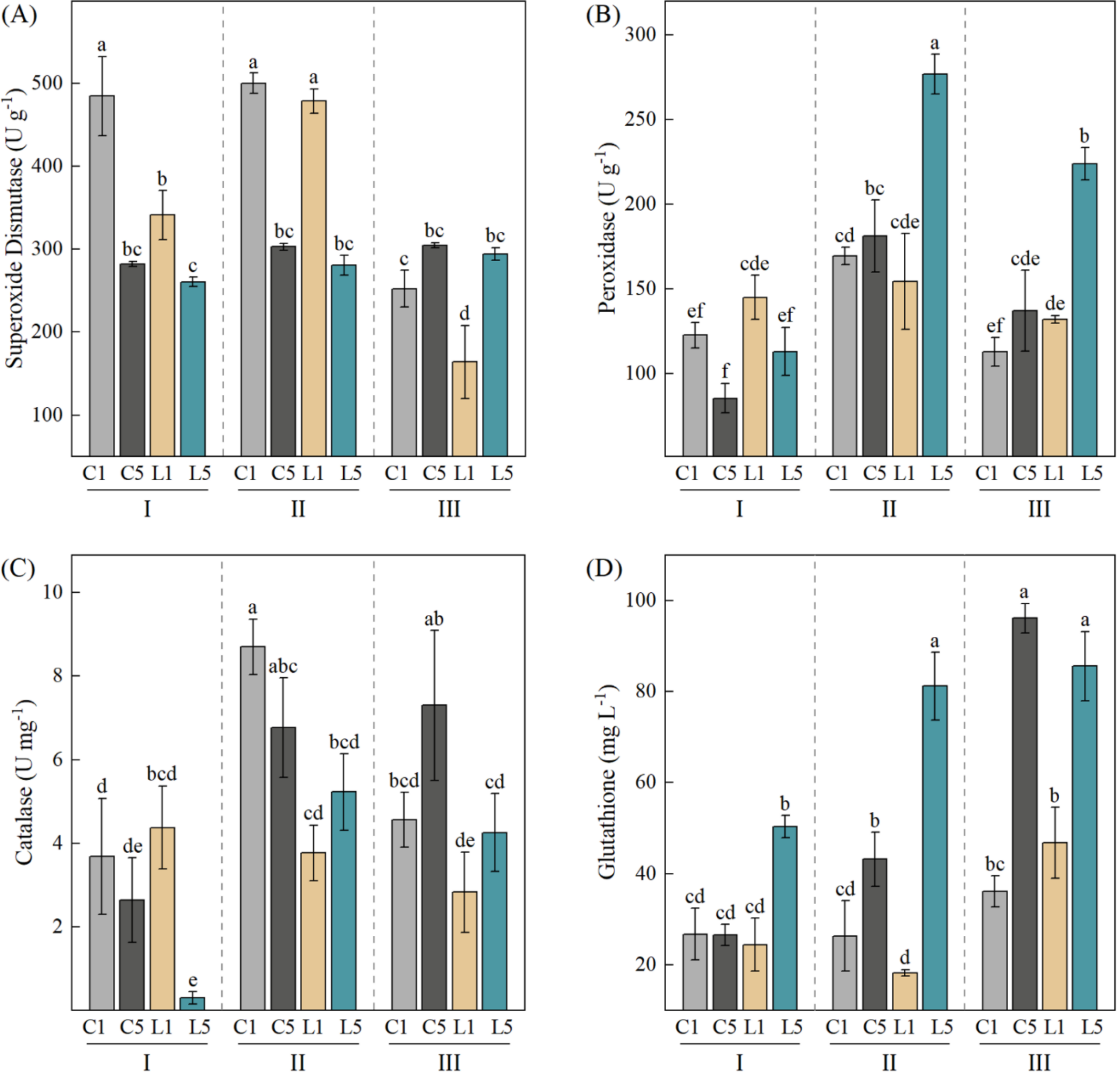
Changes in antioxidant enzyme activities of four grafting combinations under different water treatments. **(A)** superoxide dismutase; **(B)** peroxidase; **(C)** catalase; **(D)** glutathione. I, normal irrigation; II, mild water stress; III, severe water stress. C1: 'Dafeng No.1'/'Reyan No.2' (intraspecific grafting); C5: 'Reyan No.5'/'Reyan No.2' (intraspecific grafting); L1: 'Dafeng No.1'/'Charlie No.16' (interspecific grafting); L5: 'Reyan No.5'/'Charlie No.16' (interspecific grafting). Data are presented as mean ± standard deviation (n = 3). Two-way ANOVA followed by Tukey HSD test was used to determine significant differences (*p* < 0.05). Different lowercase letters indicate significant differences among treatments (*p* < 0.05).

Drought stress causes reactive oxygen species (ROS) to build up too much in plants. This triggers membrane lipid peroxidation and causes oxidative damage. Plants protect themselves by turning on their antioxidant enzyme system (SOD, POD, CAT) and building up non-enzymatic antioxidants (GSH) to clear out ROS and achieve oxidative protection (Tian et al., 2012). Two-way ANOVA showed that enzyme activity changed differently across grafting combinations as water conditions shifted. Water stress was the main factor that changed antioxidant enzyme activity in grafted coffee. The grafting combination also had a strong effect on enzyme activity (*p* < 0.001). The interaction between these two factors was strong too (**Table 4**). Under normal watering and mild stress, grafting combinations with ‘Dafeng No.1’ as the scion (C1, L1) had higher SOD activity than those with ‘Reyan No.5’ as the scion (L1, L5) (*p* < 0.05). Under severe water stress, SOD activity dropped in all four combinations. But then the pattern flipped: combinations with ‘Reyan No.5’ (L1, L5) now showed higher SOD activity than those with ‘Dafeng No.1’ (C1, L1) (**Figure 4A**). This suggests that ‘Dafeng No.1’ combinations started with higher baseline antioxidant enzyme activity. POD activity peaked in the interspecific graft L5 under mild stress (276.86 U g^-1^). This combination stayed significantly higher than others even under severe stress (*p* < 0.05) (**Figure 4B**). For CAT activity, ‘Dafeng No.1’ combinations showed higher values under mild stress than under normal watering (*p* < 0.05). The intraspecific graft C1 reached its maximum under mild stress. Under severe stress, intraspecific grafts (C1, C5) had slightly higher CAT activity than interspecific ones (L1, L5) (**Figure 4C**). Under water stress, grafting combinations with ‘Reyan No.5’ as the scion had significantly higher GSH than those with ‘Dafeng No.1’ as the scion (*p* < 0.05). The interspecific graft L5 performed well under all three conditions. Looking at the overall trend, ‘Reyan No.5’ combinations kept increasing GSH as stress got worse, while ‘Dafeng No.1’ combinations barely budged (**Figure 4D**). In summary, different grafting combinations used different antioxidant strategies to deal with water stress. SOD, POD, and CAT activity in all combinations first rose then fell as stress got worse. Under normal watering and mild stress, combinations with ‘Dafeng No.1’ as the scion had higher baseline antioxidant enzyme activity. Under severe water stress, combinations with ‘Reyan No.5’ as the scion could maintain higher enzyme activity and better reactive oxygen scavenging ability. Among the four combinations, they showed the strongest reactive oxygen scavenging ability and the best drought resistance.

**Table 4:** Results of two-way ANOVA on the effects of grafting combination (G), water treatment (W), and their interaction (G×W) on antioxidant enzyme activities (F-values).

Treatment	G	W	G×W
Superoxide Dismutase	60.84^***^	110.90^***^	48.84^***^
Peroxidase	45.71^***^	84.15^***^	18.23^***^
Catalase	13.50^***^	32.85^***^	9.24^***^
Glutathione	127.87^***^	120.10^***^	23.36^***^

Significant level: “*” represents *p* < 0.05; “**” represents *p* < 0.01; “***” represents *p* < 0.001.

### 3.5 Principal component analysis

**Figure 5:**
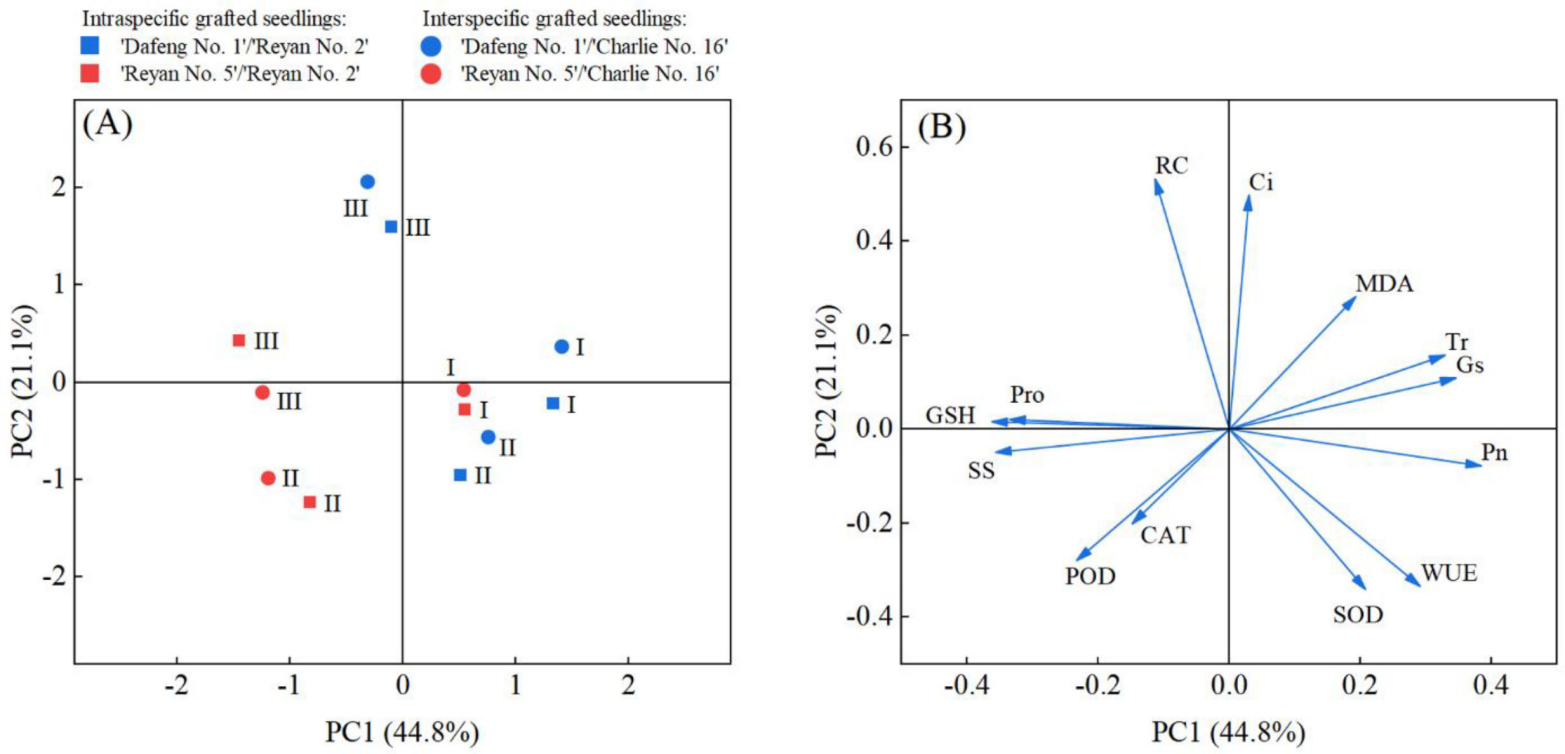
Principal component analysis (PCA) of physiological parameters in grafted coffee seedlings under different water treatments. **(A)** score plot of PCA, with points colored by scion type (blue: 'Dafeng No.1'; red: 'Reyan No.5'), shaped by grafting type (square: intraspecific grafting; circle: interspecific grafting), and labeled by water treatment (I: normal irrigation; II: mild water stress; III: severe water stress). **(B)** loading plot of PCA, showing the contribution of each physiological parameter to the principal components. Abbreviations: *P*n, net photosynthetic rate; *C*i, intercellular CO_2_ concentration; *G*s, stomatal conductance; *T*r, transpiration rate; WUE, water use efficiency; MDA, malondialdehyde; RC, relative conductivity; Pro, proline; SS, soluble sugar; SOD, superoxide dismutase; POD, peroxidase; CAT, catalase; GSH, glutathione.

We used principal component analysis (PCA) to look at how different grafting combinations responded to water stress. The results showed that PC1 and PC2 explained 44.78% and 21.09% of the total variation. Together they accounted for 65.87% of the variation (**Table 5**). In the score plot, different grafting combinations separated clearly along the PC1 axis based on scion type. Combinations with ‘Dafeng No.1’ as the scion (C1, L1) grouped mainly on the positive side of PC1. Combinations with ‘Reyan No.5’ as the scion (C5, L5) grouped mainly on the negative side. Along the PC2 axis, combinations separated by water treatment. Severe stress (III) appeared mostly in the upper part. Normal watering (I) and mild stress (II) appeared mostly in the lower part (**Figure 5A**). The loading plot showed which measurements mattered most. Photosynthesis parameters (*P*n, *G*s, *T*r, WUE) linked to the positive side of PC1. These were the main factors that set apart combinations with ‘Dafeng No.1’ scions. Osmotic adjustment and antioxidant parameters (Pro, SS, GSH, POD, CAT) linked to the negative side of PC1. These were the main factors that set apart combinations with ‘Reyan No.5’ scions. Membrane damage parameters (RC, MDA, *C*i) linked to the positive side of PC2. These were the main factors that set apart severe stress conditions (**Figure 5B**). These results show that scion type was the main factor affecting the physiological characteristics of grafted coffee plants.

**Table 5:** Eigenvalues, variance contribution rates, and cumulative variance contribution rates of principal component analysis (PCA).

Principal component number	Eigenvalue	Percentage of variance (%)	Cumulative variance (%)
1	5.82	44.78	44.78
2	2.74	21.09	65.87
3	2.18	16.75	82.62
4	0.94	7.26	89.88
5	0.69	5.28	95.16
6	0.41	3.18	98.34
7	0.11	0.83	99.17
8	0.07	0.50	99.67
9	0.03	0.26	99.93
10	0.01	0.04	99.97
11	0.00	0.03	100.00
12	0.00	0.00	100.00

PCA was performed based on the correlation matrix of physiological parameters, with all variables standardized (Z-score) before analysis. Eigenvalues represent the amount of variance explained by each principal component.

### 3.6 Correlation analysis of physiological parameters

**Figure 6:**
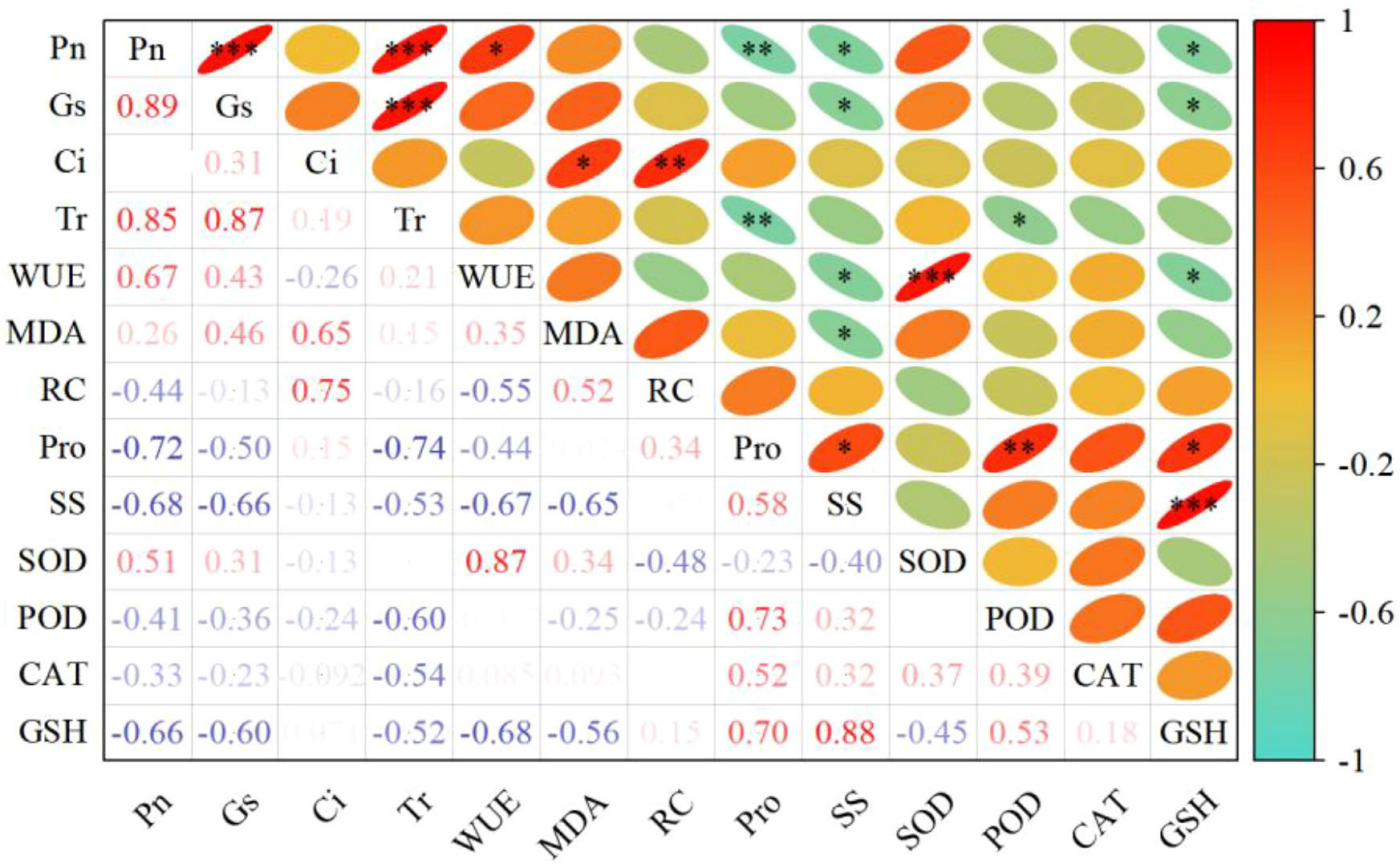
Pearson correlation heatmap of physiological parameters in grafted coffee seedlings under different water treatments. The color gradient represents the Pearson correlation coefficient, ranging from -1 (green, negative correlation) to 1 (red, positive correlation). ^*^p < 0.05; ^**^p < 0.01; ^***^p < 0.001.

We built a Pearson correlation heatmap in this study to show clearly how all the measured physiological traits relate to each other (**Figure 6**). Photosynthesis indicators showed strong positive links with each other. *P*n had very strong positive links with *G*s (r = 0.89, *p* < 0.001) and *T*r (r = 0.85, *p* < 0.001). This suggests that stomatal limitation was the main reason why photosynthetic rate dropped under water stress. Osmotic adjustment substances and antioxidant substances also showed strong positive links with each other. SS had the strongest positive link with GSH (r = 0.88, *p* < 0.001). Pro had a significant positive link with POD (r = 0.73, *p* < 0.01). These results show that the osmotic adjustment system and the antioxidant defense system work together under drought stress. It is worth noting that photosynthesis indicators had significant negative links with osmotic adjustment and antioxidant indicators. *P*n had significant negative links with Pro (r = -0.72, *p* < 0.01), SS (r = -0.68, *p* < 0.05), and GSH (r = -0.66, *p* < 0.05). WUE had a very strong positive link with SOD (r = 0.87, *p* < 0.001). This shows that SOD plays a key role in keeping WUE high under stress conditions. In summary, the correlation analysis confirms that grafted coffee seedlings face a trade-off between keeping photosynthesis going and turning on defense mechanisms. Under water stress, combinations with ‘Dafeng No.1’ as the scion focus more on keeping photosynthetic function, while combinations with ‘Reyan No.5’ as the scion give priority to turning on osmotic adjustment and antioxidant defense mechanisms.

## 4 Discussion

This study tested the idea that under severe water stress, drought resistance in grafted coffee seedlings mainly depends on the scion genotype. But under normal watering, the rootstock genotype matters more. Under normal irrigation, the interspecific rootstock *C. liberica* coffee ‘Charlie No.16’ clearly improved photosynthetic performance in both scions. However, under drought stress, all physical differences between grafting combinations were mainly driven by the scion genotype. Specifically, the scion ‘Reyan No.5’ showed much better drought resistance than ‘Dafeng No.1’ because it had stronger cell membrane stability, better osmotic adjustment capacity, and a more stable antioxidant system.

The most significant finding in photosynthetic response is the trade-off between water use efficiency under mild stress and overall drought tolerance (Rivas et al., 2016). The scion ‘Dafeng No. 1’ kept its stomata open to absorb CO_2_ under mild water stress. This helped it maintain a high net photosynthetic rate and high water use efficiency. But this strategy used too much water. Under severe stress, it caused serious cell dehydration and damage to the photosynthetic system. This matches the findings of Zekri and Lang (2024) in Arabidopsis under drought stress. In contrast, the scion ‘Reyan No. 5’ used a conservative strategy. It reduced stomatal conductance and photosynthetic rate early to save water. Because of this, its water use efficiency was lower under mild stress. But it had a higher survival rate under long-term severe drought. This is in line with the strategy found by Wang et al. (2014) in wheat. Under normal watering, the interspecific rootstock ‘Charlie No.16’ improved *P*n by 7.93% for ‘Dafeng No.1’ and 42.71% for ‘Reyan No.5’ compared with the intraspecific rootstock ‘Reyan No.2’. This shows that rootstock has a strong effect on photosynthesis under non-stress conditions. Under severe water stress, the *P*n difference between the two scions on the same rootstock was 189.58% on ‘Reyan No.2’ and 143.10% on ‘Charlie No.16’. The *P*n difference between the two rootstocks for the same scion was only 6.38% for ‘Dafeng No.1’ and 11.97% for ‘Reyan No.5’. These results clearly show that scion genotype becomes the main factor controlling photosynthetic performance under drought stress. In addition, The rise in *C*i under severe water stress mainly came from irreversible damage to the photosynthetic machinery and weaker CO_2_ fixation ability in the Calvin cycle (Reid and Kalcsits, 2020). Stronger cell respiration under stress may also push *C*i higher, but this effect was only secondary (Dias and Brüggemann, 2010).

Cell membrane stability is the most direct way to measure how well a plant can resist drought (Giannopolitis and Ries, 1977; Sedaghat et al., 2017). Under drought stress, plants produce a large amount of superoxide free radicals. These radicals cause lipid peroxidation in the cell membrane. This process produces malondialdehyde (Feng et al., 2023). At this point, the cell membrane loses its stable structure. Electrolytes leak out from the cell. This makes the RC value go up (Lin et al., 2022). Our results show that under all water conditions, the scion ‘Reyan No.5’ had significantly lower MDA content and RC than ‘Dafeng No.1’(*p* < 0.05). This difference became even more obvious under severe stress. It is worth noting that rootstock genotype had no significant effect on MDA content (*p* > 0.05). This shows that cell membrane stability in grafted coffee seedlings is completely determined by the scion genotype. PCA further confirmed that under severe stress, RC and MDA were the main physical factors that limited drought resistance in ‘Dafeng No.1’ scions. In contrast, these membrane damage indicators had less effect on ‘Reyan No.5’. This explains why ‘Reyan No.5’ showed better overall drought resistance.

Osmotic adjustment is the core drought resistance mechanism in grafted coffee (Maggio et al., 2002). Our results clearly show that this trait is mainly determined by the scion genotype. Pro and SS are the main osmotic adjustment substances that keep cell turgor stable and protect proteins and cell membranes under drought (Livingston et al., 2009; Filippou et al., 2014). In this study, both Pro and SS built up significantly as drought got worse. But the amount of buildup differed greatly between different scions (*p* < 0.05). This finding is consistent with grafting results reported in other species, such as tomato (Zhang et al., 2020) and polyploid mulberry (Hui et al., 2024). It is worth noting that ‘Reyan No.5’ had much stronger osmotic adjustment ability than ‘Dafeng No.1’. This advantage stood out even more under severe water stress. Under severe drought, both C5 and L5 combinations had significantly higher Pro and SS buildup than C1 and L1 combinations (*p* < 0.05), no matter what rootstock was used. This response directly reduces cell dehydration, keeps cell membranes intact, and ensures normal metabolic function. This stronger osmotic adjustment ability is the key physical mechanism that gives ‘Reyan No.5’ scions excellent drought resistance. PCA further confirmed that under drought conditions, Pro and SS were the main physical drivers that set apart ‘Reyan No.5’ from ‘Dafeng No.1’. In contrast, rootstock had weak and insignificant effects on osmotic adjustment under severe stress. This further shows that osmotic adjustment is a key trait determined by the scion, and it is vital for drought resistance in grafted coffee.

Different scion genotypes use completely different antioxidant strategies to deal with drought stress (Mittler, 2002; Choudhury et al., 2016). The ‘Dafeng No.1’ scion relies on higher baseline superoxide dismutase activity under normal and mild stress conditions. However, under severe stress, SOD easily loses its activity due to damage. This causes a sharp drop in reactive oxygen scavenging ability and leads to membrane lipid peroxidation (Wang et al., 2020). In contrast, the ‘Reyan No.5’ scion mainly depends on peroxidase and catalase. These two enzymes stay more stable under severe stress (Zheng et al., 2023). This difference in antioxidant strategy explains why ‘Reyan No.5’ scion confers better drought tolerance than ‘Dafeng No.1’ scion. Also, as stress gets stronger, ‘Reyan No.5’ can keep making glutathione. This further boosts its reactive oxygen scavenging ability (Lou et al., 2018). Among the four grafting combinations, the interspecific graft L5 (‘Reyan No.5’/’Charlie No.16’) showed the strongest reactive oxygen scavenging ability under severe water stress. This is because this combination has both the excellent antioxidant traits of the ‘Reyan No.5’ scion and the moderate growth-promoting effect of the ‘Charlie No.16’ rootstock.

Principal component analysis (PCA) fully supported our core idea. Under normal watering, the PCA score plot showed that grafted seedlings grouped clearly by rootstock type. This suggests that rootstock genotype has a stronger effect on plant physical traits under non-stress conditions. Under drought stress, all samples grouped clearly by scion type instead. This had nothing to do with rootstock type. This directly proves that scion genotype drives the physical response in grafted coffee seedlings under drought stress.

## 5 Conclusion

This study shows that under severe water stress, scion genotype is the main factor that decides drought resistance of grafted coffee seedlings. But under normal water supply, rootstock genotypes have a stronger effect. Specifically, the *C. canephora* scion ‘Reyan No.5’ has better overall drought resistance than ‘Dafeng No.1’. It has stronger cell membrane stability, better osmotic adjustment ability and a more stable antioxidant system. One key finding of this study clarifies the trade-off between water use efficiency and drought resistance. ‘Dafeng No.1’ gets higher WUE under mild stress by keeping a high photosynthetic rate. But this growth strategy costs too much under long-term drought. It will cause serious damage to cell membranes and make the photosynthetic system stop working. In contrast, ‘Reyan No.5’ uses a conservative strategy. It lowers stomatal conductance early to save water. It has lower WUE under mild stress, but it has a much higher survival rate under severe stress. As drought stress gets worse, the limit to photosynthesis changes from stomatal limitation to non-stomatal limitation. No matter what rootstock is used, ‘Reyan No.5’ has significantly lower malondialdehyde content and relative conductivity. This means its cell membranes suffer less oxidative damage. At the same time, this scion can build up more proline and soluble sugars. This improves its osmotic adjustment ability and keeps cell turgor stable. Antioxidant enzyme activity in all grafting combinations first rises then falls. But the interspecific graft L5 (‘Reyan No.5’/’Charlie No.16’) still has the highest reactive oxygen scavenging ability under severe stress. It is the best drought-resistant material among all tested combinations. These results give clear and practical guidance for coffee grafting breeding in dry areas. For ‘Dafeng No.1’ scions, farmers need to water more often to avoid severe water stress. For ‘Reyan No.5’ scions, moderate insufficient irrigation is acceptable. To improve the long-term drought survival ability of coffee, choosing drought-resistant scions like ‘Reyan No.5’ is more important than choosing rootstocks. Future studies should focus on the molecular mechanisms of scion-dominated drought resistance. This includes signal transmission and gene expression regulation between scions and rootstocks under continuous water stress.

## Data Availability

The original contributions presented in the study are included in the article/supplementary material. Further inquiries can be directed to the corresponding authors.
